# Distinctive Sans Forgetica font does not benefit memory accuracy in the DRM paradigm

**DOI:** 10.1186/s41235-022-00448-9

**Published:** 2022-12-09

**Authors:** Mark J. Huff, Nicholas P. Maxwell, Anie Mitchell

**Affiliations:** 1 School of Psychology, The University of Southern Mississippi, 118 College Dr. #5025, Hattiesburg, MS 39406 USA; 2 Present Address: Midwestern State University, Wichita Falls, TX USA

**Keywords:** Sans Forgetica, Associative memory errors, Free recall, Recognition, Distinctiveness

## Abstract

A common method used by memory scholars to enhance retention is to make materials more challenging to learn—a benefit termed desirable difficulties. Recently, researchers have investigated the efficacy of Sans Forgetica, a perceptually disfluent/distinctive font which may increase processing effort required at study and enhance memory as a result. We examined the effects of Sans Forgetica relative to a standard control font (Arial) on both correct memory and associative memory errors using the Deese/Roediger–McDermott (DRM) false memory paradigm, to evaluate Sans Forgetica effects on overall memory accuracy. Across four experiments, which included nearly 300 participants, Sans Forgetica was found to have no impact on correct or false memory of DRM lists relative to a standard Arial control font, regardless of whether font type was manipulated within or between subjects or whether memory was assessed via free recall or recognition testing. Our results indicate that Sans Forgetica is ineffective for improving memory accuracy even when accounting for associative memory errors.

Memory researchers are highly invested in discovering techniques that can promote memory accuracy. While dozens of strategies have been identified, including those that affect processes occurring at study and test (see Neath, 1998, for review), tasks that improve encoding processes are often focal given that they are simple to manipulate and produce reliable benefits. Effective encoding tasks often operate to enhance semantic processing of study materials. Based on the levels-of-processing framework (Craik & Lockhart, 1972), effective encoding tasks (termed “deep” processing tasks) qualitatively affect the processing of study materials which improve later correct recall and recognition. For instance, deep tasks can facilitate semantic processing of study materials and/or may enhance the distinctiveness of individual study items, making them more memorable (see Gallo et al., 2008, for review; Fisher & Craik, 1977). Deep tasks are often contrasted to “shallow” or “neutral” tasks (i.e., a read-only or intentional encoding task) which are less likely to enhance semantic or distinctive processing. While 50 + years of memory research has affirmed the advantage for deep encoding tasks (though interactions can occur with retrieval context; see Blaxton, 1989; Morris et al., 1977), the present study evaluated whether a recently developed disfluent/distinctive font type termed Sans Forgetica can benefit overall memory accuracy relative to a standard font type on correct memory and associative memory errors.

Sans Forgetica font was developed by a team of researchers from the Royal Melbourne Institute of Technology (RMIT) with the goal of producing a typeface that would aid memory retention in everyday contexts. This font is characterized by an italicized, back-slanted, and fragmented style that was suggested to encourage additional processing efforts to perceive and encode (see Fig. 1 for examples). Potential Sans Forgetica memory benefits were based on *desirable difficulties*, in which additional efforts expended at study often yield long-term memory improvements. Indeed, the memorial benefits of desirable difficulties have been well-supported by previous research (see Bjork, 1994; Bjork & Bjork, 2011; Dunlosky et al., 2013, for reviews). For instance, generating words at study either through stem-completions or solving anagrams produces correct memory benefits relative to studying words intact (Bertsch et al., 2007; Huff & Bodner, 2013; Slamecka & Graf, 1978). Similar patterns have also been reported via production (saying words aloud vs. silently; Ozubko & MacLeod, 2010; Fawcett, 2013) and drawing images of a word’s referent compared to studying the words intact (Namias et al., 2022; Wammes et al., 2016). Similarly, Rosner et al. (2015) found that blurring words, which likely makes words disfluent and more challenging to perceive, can improve memory relative to non-blurred words (but see Bjork & Yue, 2016). Collectively, additional efforts expended at encoding can facilitate memory, and these benefits manifest using different study tasks.

**Fig. 1:**
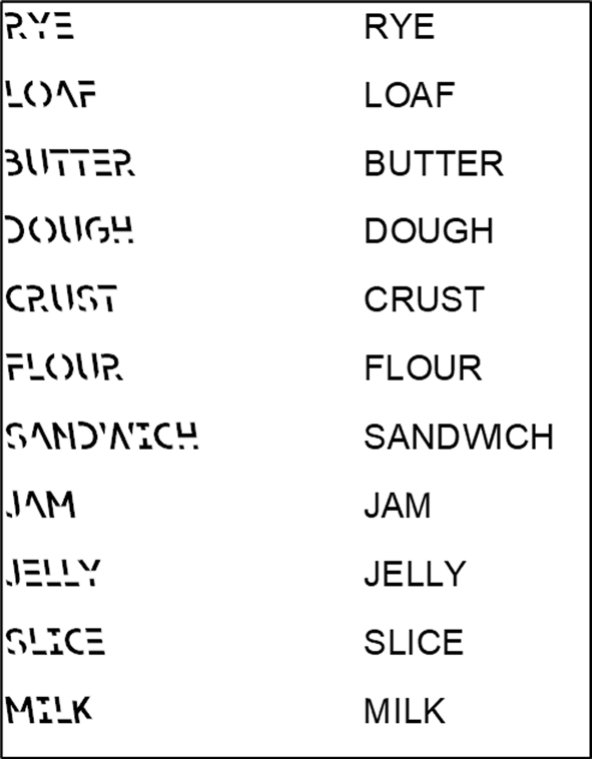
Examples of DRM lists presented using Sans Forgetica font (left) and Arial font (right)

Consistent with desirable difficulties, early evidence using Sans Forgetica font yielded memory benefits. Specifically, the RMIT team presented university undergraduates with a set of word pairs that were displayed in one of three different types of broken/disjointed formats that varied between slight, moderate, and extreme disfluency and one set of word pairs presented in a standard fluent Arial font. Pairs presented in the moderate disfluent font were better remembered than the slight and extreme disfluency formats (8% improvement) and only slightly better (1%) than the fluent font. In a second dataset collected online, the moderate disfluent font was directly compared to a standard Arial font, and a 7% memory benefit was reported (see Earp, 2018).^1^ Thus, Sans Forgetica appeared to be a method for improving memory consistent with desirable difficulties.

Although initial benefits of Sans Forgetica were encouraging for both basic and applied contexts given the relative ease in which textual font types can be adjusted for educational materials, the effects of disfluent fonts on memory are mixed. While some disfluent fonts/word presentations have been shown to produce memory benefits (e.g., Diemand-Yauman et al., 2011; Sungkhasettee et al. 2011), others produced no effects on correct memory (Arndt & Reder, 2003; Rhodes & Castel, 2008), and in some cases, have produced a memory cost (Eitel & Kühl, 2016; Kühl et al., 2014; Maxwell et al., 2022; Yue et al., 2013). A meta-analysis (Xie et al., 2018), however, indicated that perceptual disfluency in study materials, including those that were perceptually distorted, produced no effect on later recall relative to non-distorted controls, suggesting that disfluent materials may not procure benefits to correct memory.

More recently, researchers have directly compared the memory effects of Sans Forgetica words relative to words presented in a standard font type. Using a within-subject design, Taylor et al. (2020) reported that Sans Forgetica font yielded a memory cost for target memory following study of cue-target pairs and no effect of font type for cued-recall of prose passages and of educational materials when compared to a standard Arial typeface. The lack of Sans Forgetica benefits occurred despite participants rating Sans Forgetica items as being subjectively more challenging to read than materials displayed in an Arial font. Similar null effects on educational materials were echoed by Geller et al. (2020) who found that Sans Forgetica had no effect on recognition discriminability. Finally, Maxwell et al. (2022) found a Sans Forgetica cost on target recall of word pairs and, further, that participants did not expect Sans Forgetica pairs to be better remembered on a subsequent test compared to Arial pairs based on judgments of learning provided at study (cf. Geller & Peterson, 2021). Collectively, presenting study materials via Sans Forgetica font does not appear to produce a memory benefit relative to a control font and may even produce a memory cost when participants study cue-target pairs.

Despite relatively consistent findings that disfluent fonts do not procure an advantage to correct memory, they may still benefit overall memory accuracy when errors are considered. A common method for examining the effects of errors on memory accuracy is by using study materials that are conducive to commission errors such as the Deese/Roediger–McDermott (DRM; Deese, 1959; Roediger & McDermott, 1995) paradigm. In this paradigm, participants study lists of associatively related words (*cake, nice, sugar,* etc.) that are directly related to a non-presented critical lure (e.g., *sweet*). At test, participants are highly susceptible to falsely remembering the critical lure, a pattern termed the DRM illusion. False recall can eclipse 50% (Roediger & McDermott, 1995) and false recognition can approach and even exceed hit rates of studied list items (see Gallo, 2006; Huff & Bodner, 2014, for reviews; Lampinen et al., 1999). Because intrusions and false alarms are more common for associated materials, an important question is whether a disfluent font such as Sans Forgetica might reduce false memory errors despite evidence indicating that Sans Forgetica is ineffective at facilitating correct memory.

Indeed, there is reason to expect that disfluent fonts may benefit overall accuracy in the DRM paradigm through the reduction of memory errors. For instance, Arndt and Reder (2003) presented participants with DRM lists in which all words were presented using the same font or were presented such that each list word was presented in a unique font which may have disrupted the fluency of the list. Overall, correct recognition of DRM lists was equivalent between same and unique font conditions, but unique fonts reduced false recognition of critical lures, a pattern which was found using both between- and within-subject designs. When considered alongside studies using Sans Forgetica, Arndt and Reder’s findings suggest that although unique fonts may be ineffective at promoting correct recognition, they may still benefit overall memory accuracy by reducing false recognition of critical lures.

Similar fluency-related patterns have been found in studies examining the generation effect (Slamecka & Graf, 1978). In these studies, participants study DRM lists that are either presented as intact list words or are presented as disfluent anagrams in which participants must generate the word by exchanging specific letters. Across studies, generation of the disfluent anagram improves correct memory while decreasing false memory for the critical lure (Gunter et al., 2007; Huff & Bodner, 2013; Huff et al., 2020; McCabe & Smith, 2006), a pattern which has been referred to as a mirror effect (Glanzer & Adams, 1990). These disfluency effects are often ascribed as promoting distinctive or item-specific processing as participants are tasked with processing unique perceptual features of each item which likely restricts the processing of related features including the critical lure (see Hege & Dodson, 2004, for description of an impoverished relational encoding account). Moreover, encoding tasks that encourage distinctive/item-specific processing consistently reduce the DRM illusion and often increase correct recall and/or recognition. In addition to generation, these tasks include studying DRM list items that are presented with a picture of a word’s referent (Israel & Schacter, 1997; Schacter et al., 1999), creating mental images of individual words (Oliver et al., 2016; Robin, 2010), drawing images of words (Namias et al., 2022), and using study tasks such as pleasantness ratings which encourage the processing of item-specific characteristics (Huff & Bodner, 2013; Huff et al., 2015; McCabe et al., 2004). Given the similarities between perceptually disfluent manipulations of DRM lists and item-specific processing tasks, if a perceptually disfluent Sans Forgetica font reduces the DRM illusion, it may reflect the presence of item-specific processing.

In the present study, we further examined the effects of a perceptually disfluent Sans Forgetica font by examining correct and false memory using the DRM paradigm. First, in Experiments 1A and 1B, participants studied a series of DRM lists presented in either Sans Forgetica or a fluent Arial font and were then tested via free recall. Importantly, font effects were manipulated both within and between subjects (Experiment 1A and 1B, respectively). We assessed the effects of experimental design given that distinctive encoding effects such as pictorial encoding have been shown to be effective at reducing the DRM illusion in between-subject but not within-subject designs, suggesting the use of a global distinctiveness heuristic (Schacter et al., 2001; see Huff et al., 2015 for further discussion). Experiments 2A and 2B then tested whether Sans Forgetica could reduce false memory in an old/new recognition test rather than free recall. Like Experiment 1, Experiment 2 similarly tested for these effects using both within-subject (2A) and between-subject (2B) designs. Thus, any reductions in the DRM illusion due to Sans Forgetica were expected to occur regardless of whether participants completed free-recall or recognition testing, given that item-specific encoding has been shown to be effective at reducing the DRM illusion for both test types (Huff & Bodner, 2013, 2019).

Our experiments were designed to provide a comprehensive test of Sans Forgetica effects on correct memory given researchers have been unable replicate the early reported benefits of Sans Forgetica (e.g., Taylor et al., 2020; Geller et al., 2018) while also evaluating Sans Forgetica effects on false memory errors—a novel contribution. Furthermore, we examined Sans Forgetica effects using different experimental designs and test types which may serve as boundary conditions for potential Sans Forgetica benefits that have not yet been tested.

## Experiment 1A: Arial versus Sans Forgetica within-subject recall

The goal of Experiment 1A was to test whether Sans Forgetica font would benefit memory within the context of the DRM paradigm using a within-subject design. Because previous research has shown no benefit of Sans Forgetica on correct memory (e.g., Geller et al., 2020; Maxwell et al., 2022), we similarly expected no benefit in correct recall for items presented in Sans Forgetica relative to Arial font. However, given that Arndt and Reder (2003) found that presenting DRM lists using unique, distinctive fonts reduced the DRM illusion by disrupting the fluency of the list, we anticipated that Sans Forgetica would produce a similar reduction such that false recall of critical items would be lower when study lists were presented using Sans Forgetica versus Arial font.

## Methods

### Participants

Fifty-one University of Southern Mississippi undergraduates completed the study online for partial course credit. Data from 8 participants were eliminated for either failing to complete memory tests for all study lists (*n* = 4) or for perfect or near-perfect recall (i.e., recall greater than 95%) suggestive of cheating (*n* = 4). This resulted in 43 participants available for analysis in the final dataset. A sensitivity analysis using G*POWER 3 (Faul et al., 2007) indicated that the sample had adequate statistical power (0.80) to detect medium effect sizes of Cohen’s *d* = 0.44 or larger, two-tailed. Participants reported normal or corrected-to-normal color vision.

### Materials

Twenty DRM lists with the highest backward associative strength (BAS) from Roediger et al. (2001) served as study materials. Each list contained 12 total words which were presented in descending order of backward associative strength (BAS). Words were displayed for 2.5 s each. Lists were divided into two sets of 10 lists that were matched on BAS and counterbalanced across participants. List order was once randomized and presented in the same order across participants. From these two sets of lists, half were presented in a standard 32 pt. Arial font, whereas the other half were presented in a 32 pt. Sans Forgetica font. Lists alternated between Arial and Sans Forgetica font types and two additional counterbalances were created in which one version started with Arial font type such that list fonts alternated Arial, Sans Forgetica, Arial, etc. and the other started with Sans Forgetica font type and alternated Sans Forgetica, Arial, Sans Forgetica, etc. At study, lists were separated by a filler task which consisted of a word-generation task in which participants were given a letter and asked to generate as many words that begin with that letter as possible. The letter that was used for the filler tasks was also once randomized and presented in the same order across participants.

### Procedure

Participants were tested online via *Collector*, an open-source program designed to proctor web-based experiments in Psychology (Garcia & Kornell, 2015). Following informed consent, participants were instructed that they would view a series of word lists and that after each list they would complete an unspecified memory test. Participants were not informed that the lists would be related nor were they informed about the non-presented critical lures. No explicit encoding strategy was requested, and participants were not informed that the word lists would be presented in different fonts. Following the presentation of the first list, participants completed a 60-s filler task in which they were to list as many words as they could that began with a specified letter (e.g., “K”). Immediately following the filler task, participants then completed a free-recall test in which they were instructed to recall as many words from the most recent study list as they could without penalty for misspellings. They were further informed that they would have 60 s to complete the test. Following the test phase, the computer program immediately advanced to an instruction screen informing the participant that they would study another list which would be followed by another memory test. Participants repeated this cycle until all 10 DRM lists were studied and tested. Following the final test phase, participants completed a brief demographics questionnaire and were fully debriefed regarding the study. The experimental duration was less than 30 min.

## Experiment 1B: Arial versus Sans Forgetica between-subject recall

Next, Experiment 1B tested whether Sans Forgetica would affect recall in the DRM paradigm using a between-subject design. We again expected that correct recall would not differ between items presented in Sans Forgetica and Arial fonts, and further, that participants in the Sans Forgetica group would show a reduction in the DRM illusion compared to participants in the Arial group. Thus, we anticipated that effects of Sans Forgetica on false recall would not be restricted to a within-subject design.

## Methods

### Participants

One-hundred-four University of Southern Mississippi undergraduates were recruited to participate in the study for partial course credit. Participants were recruited online and were randomly assigned to either the standard-font group or the Sans Forgetica font group. Data from 10 participants were eliminated for either failing to complete memory tests for all study lists (*n* = 3), or perfect or near-perfect recall (i.e., > 95%) suggesting cheating (*n* = 7). After these participants were eliminated, 44 were available in the standard-font group, and 50 in the Sans Forgetica group. A sensitivity analysis again indicated that the sample had adequate statistical power (0.80) to detect medium effect sizes of Cohen’s *d* = 0.52 or larger, two-tailed. All participants reported normal or corrected-to-normal vision.

### Materials and procedure

Materials and experimental procedures used in Experiment 1B were identical to that of Experiment 1A with the exception that participants were only presented with either lists in a standard Arial font or a Sans Forgetica font. As a result, only the list set was counterbalanced across participants using two versions.

## Results: Experiments 1A and 1B

Proportions of correct recall of list items, false recall of critical lures, and mean number of extra-list intrusions recalled per list as a function of standard and Sans Forgetica fonts are reported in Table 1 for Experiments 1A and 1B. A *p* < 0.05 significance criterion was adopted for all analyses. For brevity, *p*-values are not reported for statistically reliable comparisons, and we instead include effect size estimates. For non-reliable comparisons, a supplemental test using a Bayesian estimate of the strength supporting the null hypothesis was conducted (Masson, 2011; Wagenmakers, 2007). In this analysis, a model which assumes a null effect is compared to a model which assumes an effect. A *p*-value is then computed (termed *p*_BIC_; Bayesian Information Criterion), which provides an estimate of the probability that the null hypothesis is retained. The *p*_BIC_ statistic does not require the specification of priors which can be used subjectively to determine the outcome of other Bayesian methods such as Bayes factors. In addition, *p*_BIC_ does not rely upon cutoffs for “strength of evidence” for the null hypothesis. Moreover, *p*_BIC_ is highly sensitive to the sample size, in which large samples increase the confidence in a null effect. Because Sans Forgetica font often results in null comparisons relative to a standard/fluent font, we supplement null effects with this Bayesian analysis to increase confidence in the reliability of the results.

**Table 1:** Mean (± 95% CI) proportions of correct and false recall and “old” recognition responses and signal detection indices for lists presented in Arial font and Sans Forgetica font in Experiments 1 and 2

List type	Experiment 1A		Experiment 1B	
	Within recall		Between recall	
	Arial lists	Sans Forgetica lists	Arial lists	Sans Forgetica lists
*N*	43		44	50
Correct recall	.53 (.04)	.54 (.04)	.59 (.04)	.56 (.03)
False recall	.27 (.07)	.32 (.07)	.27 (.06)	.31 (.05)
# Intrusions per list	0.27 (.10)	0.26 (.12)	0.26 (.08)	0.22 (.07)

List type	Experiment 2A		Experiment 2B	
	Within recognition		Between recognition	
	Arial lists	Sans Forgetica lists	Arial lists	Sans Forgetica lists
*N*	45		58	58
List items	.70 (.05)	.70 (.05)	.70 (.04)	.66 (.04)
List item controls	.21 (.04)		.21 (.04)	.21 (.04)
List item *d*′	1.58 (.26)	1.58 (.24)	1.57 (.21)	1.42 (.18)
List item λ	0.97 (.18)		0.97 (.17)	0.96 (.17)
Critical items	.66 (.08)	.65 (.08)	.70 (.06)	.66 (.06)
Critical item controls	.24 (.06)		.29 (.06)	.33 (.06)
Critical item *d*′	1.19 (.24)	1.16 (.24)	1.27 (.22)	0.99 (.21)
Critical item λ	0.75 (.17)		0.66 (.19)	0.54 (.19)

Correct recall was found to be equivalent for lists presented in both a standard Arial font and in the Sans Forgetica font both when font type was manipulated within subjects in Experiment 1A (0.53 vs. 0.54, for Arial and Sans Forgetica fonts, respectively), *t* < 1, *p*_BIC_ = 0.86, and when font type was manipulated between subjects in Experiment 1B (0.59 vs. 0.56), *t*(42) = 1.13, *SEM* = 0.03, *p* = 0.26, *p*_BIC_ = 0.83. This equivalence extended to false recall of critical lures where Arial and Sans Forgetica fonts produced equivalent rates both in Experiment 1A (0.27 vs. 0.32), *t*(42) = 1.13, *SEM* = 0.04, *p* = 0.26, *p*_BIC_ = 0.77, and in Experiment 1B (0.27 vs. 0.31), *t* < 1, *p*_BIC_ = 0.86. Finally, mean numbers of extra-list intrusions per list (i.e., other intrusions that were not the critical lure) were rare, and did not differ between Arial and Sans Forgetica fonts in either Experiment 1A (0.27 vs. 0.26), *t* < 1, *p*_BIC_ = 0.86, or Experiment 1B (0.26 vs. 0.22), *t* < 1, *p*_BIC_ = 0.88. Thus, Sans Forgetica font type had no effect on correct or false recall in either within- or between-subject contexts.

## Discussion

Experiments 1A and 1B tested the effects of Sans Forgetica font on correct and false recall using the DRM paradigm. In doing so, we tested whether the potentially distinctive nature of Sans Forgetica would (1) improve correct recall for studied items relative to Arial font and (2) would reduce the DRM illusion by lowering false recall of non-presented critical items. Consistent with our predictions, correct recall did not differ between items presented in Sans Forgetica or Arial fonts, regardless of whether font type was manipulated within or between subjects. Similarly, false recall of non-presented critical items did not differ between font types. Thus, Sans Forgetica font was ineffective at reducing the DRM illusion relative to Arial font.

Our finding that correct recall did not differ as a function of font type is consistent with previous research showing no correct memory benefit of Sans Forgetica when compared to a more perceptually fluent font (e.g., Geller et al., 2020; Maxwell et al., 2022; Taylor et al., 2020). Additionally, our extension of this null pattern to false recall provides further evidence that Sans Forgetica is not effective at improving overall memory accuracy. However, given that distinctive encoding manipulations have been shown to be effective at reducing the DRM illusion when recognition testing is used (e.g., Huff & Bodner, 2013, 2019), it may be the case that Sans Forgetica would be effective at reducing the illusion for this test type. Experiments 2A and 2B were designed to test this possibility, again using between- and within-subject designs.

## Experiment 2A: Arial versus Sans Forgetica within-subject recognition

The primary goal of Experiments 2A was to test whether Sans Forgetica font would reduce the DRM illusion on recognition. Like Experiments 1A and 1B, we expected that Sans Forgetica would produce no benefit on correct recognition, given that previous research by Geller et al. (2020) showed that null effects of Sans Forgetica versus Arial fonts extend to recognition tests. Recognition testing, however, may be more sensitive towards detecting Sans Forgetica effects, provided Sans Forgetica is promoting distinctive/item-specific processing. Indeed, free-recall tests benefit from improved organization which is promoted more via relational encoding than item-specific encoding. In contrast, items in recognition tests are often presented randomly and therefore may be more sensitive to distinctive/item-specific encoding manipulations (Huff & Bodner, 2014; McDaniel et al., 1988). Therefore, we expected that recognition testing would be more sensitive at detecting Sans Forgetica effects on memory, particularly on false recognition which is sensitive to distinctive/item-specific processing (Huff & Bodner, 2013).

Our adoption of recognition tests also allowed for the application of a signal detection analysis to provide estimates of both encoding and monitoring processes (see Huff et al., 2015, for an in-depth discussion of applying signal detection to the DRM paradigm). Signal detection attempts to separate memory experiences for studied and non-studied items from bias, or the relative tendency to report that a test item was studied. Using this analysis, we generate two parameter estimates. The first parameter is discriminability (or *d*′) which refers to the standardized mean distance between the hit rate and false alarm distributions. We interpret *d*′ as an index of the amount of memory information encoded for a particular item type. This parameter can also be extended to DRM critical lures in which false alarms to critical lures are treated as hits and are compared to false alarms to critical lure controls (i.e., DRM critical lures from lists that were not studied). This analysis can therefore provide an estimate of the amount of memory information encoded for studied list items and DRM critical lures. The second parameter is a bias measure termed lambda (*λ*), which is computed as the *z*-score of 1 minus the false alarm rate to control items. Higher lambda estimates suggest a more conservative response bias, which we interpret as evidence for more (vs. less) test-based monitoring. To provide a more comprehensive assessment of Sans Forgetica effects on recognition, we provide signal detection estimates to accompany standard hit and false alarm recognition analyses.

## Methods

### Participants

Fifty-three University of Southern Mississippi undergraduates completed the study online for partial course credit. Data from 8 participants were eliminated due to excessive false alarm rates to non-studied control items (> 90%), indicating that participants were repeatedly pressing the “old” key and were not following directions. Forty-five participants were available for analysis. A sensitivity analysis again indicated that the sample had adequate statistical power (0.80) to detect small-to-medium effect sizes of Cohen’s *d* = 0.38 or larger, two-tailed. All participants reported normal or corrected-to-normal color vision.

### Materials and procedure

All study materials and procedures were the same as those used in Experiment 1A with the following exceptions. First, the recall test was replaced with an 80-item old/new recognition test in which all items were presented in the standard 32-pt. Arial font. The test was composed of 30 list items (15 from Arial and Sans Forgetica list types) taken from presented study lists (positions 2, 8, and 10), 10 critical lures from studied lists (5 from standard and Sans Forgetica list types), 30 list item controls taken from the counterbalanced set that was not studied (from the same positions as the list items), and 10 critical lure controls taken from the non-studied set. Participants studied all 10 lists back-to-back with an instruction screen in between indicating that a new list would be presented. Participants did not complete a filler task between lists. After the final list was presented, participants were informed that they would complete an old/new recognition test in which a test item would be presented on the center of the screen and they were to use their mouse to click on the “old” button if the word was studied, and the “new” button if the word was not studied. Participants were encouraged to respond as quickly as possible but not to compromise accuracy. Following the recognition test, participants completed the same brief demographics questionnaire and debriefing as Experiment 1A.

## Experiment 2B: Arial versus Sans Forgetica between-subject recognition

Experiment 2B tested whether Sans Forgetica font would reduce the DRM illusion for recognition testing when font-type was manipulated between subjects. Like Experiment 2A, we again expected that correct recognition of list items would not differ between items presented in Sans Forgetica and Arial fonts. Furthermore, false recognition of critical items was expected to be reduced for lists presented in Sans Forgetica font. Thus, we expected that Sans Forgetica would not be an effective means of increasing correct recognition, but would reduce DRM false recognition in both within- and between-subject contexts.

## Methods

### Participants

An additional 124 University of Southern Mississippi undergraduates completed the study for partial course credit. Participants were randomly assigned to either the standard-font group, or the Sans Forgetica font group. Data from 8 participants were eliminated due to either excessive false alarms to non-studied control items (> 90%; *n* = 5), or due to excessive misses on studied list items (hit rates < 10%), the latter of which suggests that participants were repetitively pressing the “new” button. In both cases, participants likely did not follow study instructions.^2^ Of the remaining participants, 58 were in the standard-font group and 58 were in the Sans Forgetica font group. A sensitivity analysis indicated that the sample had adequate statistical power (0.80) to detect medium effect sizes of Cohen’s *d* = 0.46 or larger, two-tailed. Again, all participants had normal or corrected-to-normal vision.

### Materials and procedure

The same materials and procedure from Experiment 2A, including the recognition test, were used. The only difference was that, like Experiment 1B, participants only studied items from one list type (either standard Arial font or Sans Forgetica font).

## Results: Experiments 2A and 2B

Like Experiment 1, a *p* < 0.05 level of significance was adopted for all reported analyses. For the signal detection analyses, false alarm rates of 0 and hit rates of 1 were adjusted using Macmillan and Creelman’s (1991) 1/2*n* correction. Mean proportions of correct recognition of list items, false recognition of critical lures, and their corresponding signal detection indices are reported in Table 1.

For correct recognition, an index of discriminability (*d*′) was computed by taking the *z*-score of the hit rate for studied items minus the *z*-score of the false alarm rate for list item controls. For false recognition, *d*′ was similarly computed, but false alarms to critical lures were treated as hits and false alarms to critical lure controls were subtracted. Memory monitoring was also computed (*λ*), which was calculated by taking the *z*-score of 1 minus the false alarm rate to list item controls to estimate correct recognition monitoring, and the *z*-score of 1 minus the false alarm rate to critical lure controls to estimate false recognition monitoring (cf. Huff & Bodner, 2013).

Starting with correct recognition of studied list items in Experiment 2A (within subjects), Arial and Sans Forgetica fonts were found to be similar in both in raw hit rates (0.70 vs. 0.70), *t* < 1, *p*_BIC_ = 0.87, and in estimates of *d*′ (1.58 vs. 1.58), *t* < 1, *p*_BIC_ = 0.87. A similar pattern was found in between-subject groups in Experiment 2B where again, hit rates were equivalent between Arial and Sans Forgetica font types (0.70 vs. 0.66), *t*(114) = 1.28, *SEM* = 0.03, *p* = 0.20, *p*_BIC_ = 0.83, and in *d*′ (1.57 vs. 1.42), *t*(114) = 1.03, *SEM* = 0.14, *p* = 0.31, *p*_BIC_ = 0.86. Given the between-subject design in Experiment 2B, estimates of memory monitoring (*λ*) were computed for correct recognition in both font types.^3^ Monitoring, however, was also equivalent between Arial and Sans Forgetica fonts (0.97 vs. 0.96), *t* < 1, *p*_BIC_ = 0.91.

Turning to false recognition of critical lures, in Experiment 2A, standard and Sans Forgetica fonts again produced equivalent false recognition (0.66 vs. 0.65), *t* < 1, *p*_BIC_ = 0.87, and equivalent *d*′ rates (1.19 vs. 1.16), *t* < 1, *p*_BIC_ = 0.86. In Experiment 2B, false recognition of critical lures was similar between Arial and Sans Forgetica fonts (0.70 vs. 0.66), *t*(114) = 1.09, *SEM* = 0.04, *p* = 0.28, *p*_BIC_ = 0.86, as was *d*′ (1.27 vs. 0.99), *t*(114) = 1.84, *SEM* = 0.15, *p* = 0.07, *p*_BIC_ = 0.66, though this latter comparison was marginally significant, the pattern is in the direction of a Sans Forgetica advantage. Finally, standard and Sans Forgetica fonts also yielded equivalent memory monitoring for critical lures (0.65 vs. 0.54), *t* < 1, *p*_BIC_ = 0.88.

## Discussion

The results of Experiments 2A and 2B are quite clear. First, consistent with our findings in Experiment 1 as well as other studies showing Sans Forgetica to be ineffective at promoting correct memory (e.g., Geller et al., 2020; Maxwell et al., 2022), Sans Forgetica produced no benefit on correct recognition relative to list items presented in Arial font. Second, Sans Forgetica was ineffective at reducing the DRM illusion on recognition as false recognition of critical lures did not differ between Sans Forgetica and Arial lists. Also consistent with Experiment 1, null effects of Sans Forgetica held regardless of whether font was manipulated within or between subjects. Thus, it is evident that Sans Forgetica is ineffective at reducing the DRM illusion.

## General discussion

Sans Forgetica is a perceptually disfluent font designed to improve retention via desirable difficulties for real-world application. Recently, however, the benefits of this font on learning have come into question. Although previous research suggests that Sans Forgetica is not effective at benefitting correct memory (e.g., Geller et al., 2020; Maxwell et al., 2022; Taylor et al., 2020), the present study tested whether the distinctive nature of this font would be beneficial at improving memory accuracy within the DRM paradigm. Specifically, we assessed whether Sans Forgetica could reduce the DRM illusion by reducing false recall/recognition of critical items. The present study therefore provided an additional method for testing the efficacy of Sans Forgetica, as previous research has only assessed this font within the context of correct memory for studied items. Each experiment provided a further test of whether Sans Forgetica would be beneficial to retention of studied items within the context of recall and recognition testing (Experiments 1 and 2, respectively). Thus, in addition to testing the effects of Sans Forgetica on the DRM illusion, our experiments also provided additional opportunities to replicate previous work showing Sans Forgetica does not promote memory for studied items.

Overall, Sans Forgetica consistently failed to improve correct memory for studied items, as proportions of correctly remembered list items did not differ between Sans Forgetica and Arial lists, regardless of whether participants were tested via free-recall (Experiments 1A and 1B) or recognition testing (Experiments 2A and 2B). Our experiments therefore replicate previous work showing Sans Forgetica does not produce a memorial benefit compared to an Arial control font while extending these findings to include associative word lists as opposed to cue-target pairs (e.g., Geller et al., 2020, Experiment 1; Maxwell et al., 2022). Importantly, the present study also showed that Sans Forgetica was ineffective at reducing the DRM false memory illusion. Across experiments, proportions of falsely recalled/recognized critical items did not differ between lists encoded via Sans Forgetica or Arial font. Furthermore, these null effects of font-type were observed regardless of whether fonts were manipulated within subjects (Experiments 1A and 2A) or between subjects (Experiments 1B and 2B). Comparisons of signal detection parameters for encoded memory information (*d*′) and test-based memory monitoring (*λ*) were similarly equivalent between the two fonts, indicating that underlying memory processes in recognition are also not sensitive to font differences. Thus, the present study replicated previous research showing no benefit of Sans Forgetica on correct memory while subsequently extending this finding to include false memories within the DRM paradigm.

Our repeated finding that Sans Forgetica was ineffective at benefitting correct recall/recognition of list items is consistent with previous research showing this font is ineffective at promoting later retention. Previous research has commonly reported no memorial benefits (and even memorial costs) for material encoded using Sans Forgetica relative to standard fonts such as Arial. For example, Taylor et al. (2020) recently showed that Sans Forgetica produced no memory benefits when this font was applied to text passages, and additionally, showed that this font produced a memory cost on recall of cue-target word pairs. Similarly, Geller et al. (2020) found Sans Forgetica to be ineffective at improving both cued-recall and recognition memory. Finally, Maxwell et al. (2022) similarly showed that Sans Forgetica did not benefit recall of cue-target pairs and, instead, produced a memory cost. Furthermore, participants’ judgments of learning did not differ between cue-target pairs presented in Sans Forgetica or Arial. Taken together, it is evident that Sans Forgetica is not beneficial to memory, and furthermore, participants do not appear to expect that Sans Forgetica will facilitate later remembering.

While our findings are consistent with previous research showing no benefit of Sans Forgetica on retention of studied items, a novel finding from the present study is that this font is similarly ineffective at reducing false memories in the DRM paradigm. As previous research has shown that a variety of distinctive encoding measures including generation (Gunter et al., 2007; McCabe & Smith, 2006), drawing (Namias et al., 2022; Wammes et al., 2016), and, importantly, font manipulations (Arndt & Reder, 2003) are effective at reducing false memories within the DRM paradigm, we reasoned that the distinctive and disfluent nature of Sans Forgetica would similarly reduce false memories relative to a control font. At first glance, our results appear discrepant with Arndt and Reder who reported that presenting DRM list words in different fonts (vs. the same font) reduced the DRM illusion. However, it is important to clarify that Arndt and Reder’s unique font conditions presented each DRM list word in a different font that was not shared with any other words within the list. In contrast, while we reasoned that Sans Forgetica would be a distinctive type of font, all words within a given list were presented using the same typeface (i.e., Sans Forgetica or Arial), with fonts only differing between DRM lists (Experiments 1A and 2A) or between participants (Experiments 1B and 2B). Therefore, font manipulations may still be effective at reducing the DRM illusion, but lists cannot simply use a “distinctive” or “disfluent” type font for all words, as each word may need to be presented using a unique font.

Collectively, our findings that Sans Forgetica yields no benefits on correct or false memories within the DRM paradigm provide further evidence that this font is not beneficial for learning. While Sans Forgetica is purported by its developers to improve retention via desirable difficulties, it appears that either the disfluent nature of this font does not produce sufficient difficulties necessary to trigger a memory improvement or any encoding difficulties of this font are simply not desirable for learning. Although desirable difficulties have been shown to occur in a variety of contexts (see Bjork & Bjork, 2020, for review), it is not always clear what level of task difficulty or task engagement is necessary to facilitate retention (e.g., McDaniel & Butler, 2010). For instance, the effects of desirable difficulties on learning have been shown to be moderated by individual differences in intelligence (Wenzel & Reinhard, 2019), such that only average and highly intelligent individuals benefit from learning difficulty. More relevant to the current study, Eskenazi and Nix (2021) reported that Sans Forgetica can aid in the learning of spelling and word definitions, but only for individuals with naturally high spelling and reading abilities (1 *SD* above the mean). Further, Sans Forgetica benefits might be moderated by test expectancy processes. Geller and Peterson (2021) reported a Sans Forgetica benefit using a large sample, but only when participants were not expecting an upcoming memory test. Collectively, while Sans Forgetica might not produce general benefits to free recall and recognition as our study replicates and extends several prior studies, benefits may still emerge for certain individual difference factors that are related to long-term episodic memory (e.g., attentional control, working memory capacity, etc.) and different types of study contexts such as knowledge of an upcoming test. While the pragmatics of using a memory-enhancing font are highly appealing—particularly in educational contexts in which written verbal materials are common—the use of Sans Forgetica does not appear to aid learning beyond a standard font type.

Given that Sans Forgetica font does not appear to facilitate learning broadly (aside from some possible individual differences and expectancy contexts), an interesting question is *why* does Sans Forgetica font fail to promote memory? As reviewed above, disfluent words generally do not appear to aid memory (Xie et al., 2018) which suggests that Sans Forgetica may not be unique from other types of disfluency manipulations used by researchers. While there are some exceptions to this general trend, we highlight that task manipulations such as generation produce robust benefits on memory, particularly in a within-subject design (Bertsch et al., 2007). An obvious explanation for the lack of Sans Forgetica benefits is that the font type is simply not sufficiently disfluent to produce a desirable difficulty. However, an alternate account may be that the detection of disfluency itself is not beneficial for memory, but rather memory benefits require the *resolution* of disfluency. For instance, generation experiments require participants not only to perceive a disfluent stimulus, but to resolve the disfluency by producing a fluent stimulus (e.g., an anagram solution). Perhaps if participants could adjust the font style of Sans Forgetica items to a more readable/fluent font (either by changing the font or mentally visualizing a fluent font), a memory benefit would emerge compared to items in which participants adjust from a fluent font to another fluent font. While a disfluency resolution process is merely speculative, determining why disfluency can aid memory under generation-type conditions but not when reading specific typefaces could be important for understanding fluency effects on memory.

An additional possibility, suggested by an anonymous reviewer, is that increasing the delay between study and test might increase the effort needed to encode study materials, which in turn, may increase the likelihood of producing a desirable difficulty. In the current experiments, we used a short delay of 60 s to ensure that participants were not relying upon active information in short-term memory when making their retrievals. Increasing the delay between study and test, which would make retrieval more challenging, might increase the likelihood of a Sans Forgetica benefit on memory. While we did not make use of a delay in our study, we note that under recall testing conditions, which are recollection heavy and thus objectively more challenging than recognition testing, we did not find a Sans Forgetica pattern. It is therefore possible that increasing the delay, which may make retrieval more difficult, might not be a factor of when a Sans Forgetica benefit is found. Regardless, our experiments provide a strong test of when Sans Forgetica may affect memory accuracy using different experimental designs, different test types, and correct and false memory. The consistency of our experiments largely replicates patterns found by other researchers by showing no benefits of Sans Forgetica on correct memory, and further, we show that design and test type conditions are not boundaries for when Sans Forgetica effects emerge.

## Conclusion

In sum, the present study tested the effects of Sans Forgetica on correct memory while also assessing whether this font would be used to improve memory accuracy in the DRM paradigm by reducing false recall and recognition. Across four experiments, we replicated existing research showing that Sans Forgetica produced no benefit on correct recall/recognition of list items compared to Arial font, regardless of whether font-type was manipulated between or within subjects (e.g., Geller et al., 2020; Maxwell et al., 2022; Taylor et al., 2020). Additionally, we showed that Sans Forgetica produced no benefits on overall DRM accuracy, as false memory occurrences similarly did not differ between fonts. Thus, the present study adds to the existing literature showing Sans Forgetica is not an effective tool for promoting retention.

## Data Availability

Data used in all reported analyses are available via OSF (https://osf.io/AM5RB/).

## Footnotes

1 Although Earp (2018) reported means for each of the font-type conditions, inferential statistics were not available. Statistical reliability between font types could therefore not be determined in either the initial study or the online sample which compared Sans Forgetica directly to the Arial font.

2 In Experiment 2A, we similarly applied the same excessive miss criteria applied in Experiment 2B, but no participants showed excessive misses and therefore were not excluded for this reason.

3 Note that monitoring estimates cannot be computed separately for Arial and Sans Forgetica font types using a within design in Experiment 2A. This is because monitoring is computed by using the false alarm rate to non-studied control items which are not yoked to a specific font type. An overall monitoring estimate was computed based on the false alarm rate to control items which is presented in Table 1.
